# Protocol for systematic review of school-based interventions to prevent and control obesity in African learners

**DOI:** 10.1136/bmjopen-2016-013540

**Published:** 2017-03-27

**Authors:** Theodosia Adom, Thandi Puoane, Anniza De Villiers, André Pascal Kengne

**Affiliations:** 1 Nutrition Research Centre, Radiological and Medical Sciences Research Institute, Ghana Atomic Energy Commission, Legon, Ghana; 2 School of Public Health, Faculty of Community and Health, University of the Western Cape, Bellville, South Africa; 3 Non-communicable Disease Research Unit, South African Medical Research Council, Tygerberg, South Africa; 4 University of Cape Town, Cape Town, South Africa

**Keywords:** EPIDEMIOLOGY, NUTRITION & DIETETICS

## Abstract

**Introduction:**

The increasing prevalence of obesity and overweight in childhood in developing countries is a public health concern to many governments. Schools play a significant role in the obesity epidemic as well as provide favourable environments for change in behaviours in childhood which can be carried on into adulthood. There is dearth of information on intervention studies in poor-resource settings. This review will summarise the available evidence on school-based interventions that focused on promoting healthy eating and physical activity among learners aged 6–15 years in Africa and to identify factors that lead to successful interventions or potential barriers to success of these programmes within the African context.

**Methods and analysis:**

This protocol is developed following the guidelines of PRIMSA-P 2015. Relevant search terms and keywords generated from the subject headings and the African search filter will be used to conduct a comprehensive search of MEDLINE (PubMed), MEDLINE (EbscoHost), CINAHL (EbscoHost), Register Academic Search Complete (EbscoHost) and ISI Web of Science (Science Citation Index) for published literature on school-based interventions to prevent and control obesity in learners in Africa. Grey literature will be also be obtained. The searches will cover 1 January 2000 to 30 June 2016. No language limitations will be applied. Full-text articles of eligible studies will be screened. Risk of bias and quality of reporting will be assessed. Data will be extracted, synthesised and presented by country and major regional groupings. Meta-analysis will be conducted for identical variables across studies, where data allow. This protocol is developed following the guidelines of PRISMA-P 2015.

**Ethics and dissemination:**

No primary data will be collected hence ethics is not a requirement. The findings will be submitted for publication in peer-reviewed journals, in conferences and in policy documents for decision-making, where needed.

**Strengths and limitations of this study**

- The systematic approach will summarise the current available evidence on characteristics, outcomes and effectiveness of school-based interventions in Africa.
- The findings could identify potential factors that lead to successful interventions or barriers to the successful implementation of these programmes within the African context as well as serve as a policy guide to the design and implementation of effective strategies.
- Application of the GRADE approach in rating the quality of evidence will strengthen the review.
- Since only studies that used body mass index and/weight changes as body composition changes will be included, some potentially relevant studies that used other measures of body composition are likely to be excluded.

## Introduction

The increasing prevalence of obesity and overweight in childhood in developing countries is a public health concern to many governments. Childhood obesity is not only a major risk factor for obesity in adulthood,^1^ ^2^ but also increases the risk of developing hypertension, high cholesterol, orthopaedic problems and type 2 diabetes even in young children.^3^ Obese children suffer from negative psychological consequences including poor self-esteem, depression, anxiety and stigmatisation.^4^

The aetiology of obesity is multifactorial, involving individual and environmental factors. Among the individual determinants are energy expenditure and energy intake. Obesity occurs when energy intake exceeds energy expenditure over a prolonged period of time.^5^ The environmental factors also present the opportunity to engage in healthy or unhealthy behaviours. Interventions to prevent and control obesity in childhood should therefore be multicomponent targeting modifiable individual factors as well as the settings in which the interventions are implemented. In general, most interventions have focused on health education and promotion to increase knowledge, attitudes and behaviours. These interventions target the individuals by focusing on nutrition or physical activity either separately or combined, with modest effects on behavioural change and body mass index (BMI).^6^ In recent years, interventions have been directed at changing the environments related to nutrition and physical activity so as to improve healthy behaviours.^7^

Schools play a significant role in the obesity epidemic^8–10^ as well as provide favourable environments for change in behaviours in childhood which can be carried on into adulthood.^11–13^ This is because schools offer continuous and intensive contact with children coupled with the schools' organisational structures through which these interventions could be effectively delivered.^14^ ^15^ Although considerable scientific literature exists on the importance of school-based interventions to address childhood obesity in developed countries,^12^ ^15^ there is dearth of information in poor-resource countries mostly attributed to lack of funds to effectively conduct and evaluate such intervention studies. Should there be funding, competing interests in investing into other areas of health promotion instead of promoting physical activity and healthy eating might also play a role. Some studies highlight inconclusive and conflicting results while others report minimal positive weight-related outcomes and modest behaviour changes.^11^ ^15^ ^16^ These inconsistencies may be mostly due to the variability of methods used.

The increasing prevalence of obesity in African learners calls for interventions. Evidence-based strategies are critical to the success of intervention programmes; therefore, the need to synthesise available evidence in the African context. This systematic review will summarise the evidence on school-based interventions targeted at improving diet and physical activity, to gain better understanding of what intervention programmes work in the prevention and control of childhood obesity among African learners and to identify gaps in the literature for further research.

## Objective

To conduct a systematic review of the published literature to identify and characterise school-based interventions that focused on promoting healthy eating and physical activity among learners aged 6–15 years in Africa to prevent childhood obesity, as well as to identify factors that lead to successful interventions or potential barriers to success of these programmes within the African context, as reported in studies published between January 2000 and June 2016.

## Review questions

1. What are the characteristics of school-based interventions targeted at the prevention and control of childhood obesity among learners in Africa?
2. What school-based programmes are effective in promoting healthy eating and physical activity behaviours of learners?
3. What factors influence the success of these school-based programmes and what are the potential barriers to their successful implementation?

## Methods

This protocol is developed following the guidelines of PRISMA-P 2015.^17^

### Inclusion criteria

To guide selection of studies, the Population, Intervention, Comparison and Outcome (PICO) protocol^18^ will be used as outlined below:

1. *Population*: Studies involving learners aged 6–15 years of both sexes of African populations residing in African countries, or studies presenting data specifically for the subgroup of participants within 6–15 years age range.
2. *Intervention*: Primary research evaluating dietary/nutrition interventions alone, physical activity interventions alone, combined dietary and physical activity interventions, school environment.
3. *Outcome*: Studies reporting changes in nutrition and physical activity knowledge, attitude and self-efficacy, increased participation in physical activity, increased intake of fruits and vegetables, decreased consumption of high fat diets and sugar-sweetened beverages, changes in body weight or BMI-for-age and reporting a baseline and a postintervention measurements.
4. *Context*: The primary focus of interventions must be the school setting. However, school-based studies with community-based or family-based components in addition will be included.
5. *Study design*: Obesity prevention and treatment interventions that used a controlled study design with or without randomisation, pre-experimental, quasi-experiment, experimental pre-test/post-test design, cohort study design, of at least 12 weeks duration.
6. All published and unpublished studies between 1 January 2000 and 30 June 2016. The focus of research in African children had been and is still on malnutrition. Post-2000 studies are more likely presentative of the contemporary pictures as obesity and overweight are emerging public health problems in childhood which may occur alongside undernutrition, the issue of double burden of nutrition.
7. Interventions involving human participants.
8. No language limitations will be applied.

### Exclusion criteria

1. Studies that are clinic-based or have no school-based components.
2. Interventions in African populations but residing outside Africa.
3. Interventions in learners with eating disorders, critical illness or chronic conditions.
4. Interventions in learners with physical and mental disabilities.

### Search strategy

A systematic search of the following electronic databases of peer-reviewed journal articles and online search registers will be conducted using the African search filter:^19^ MEDLINE (PubMed), MEDLINE (EbscoHost) CINAHL (EbscoHost), Academic Search Complete (EbscoHost) and ISI Web of Science (Science Citation Index). Search terms will include key words relating to population (learners, ‘schoolchildren’, ‘school going children’); interventions: (diet-related, physical activity-related, school environment-related); geographical settings (African search filter) and outcomes (changes in nutritional and physical activity knowledge, attitude and self-efficacy, increased participation in physical activity, increased intake of fruits and vegetables, decreased consumption of high fat diets and sugar-sweetened beverages, changes in body weight or BMI-for-age). The search terms will be combined to suit each database. A search strategy for PubMed database is attached (see online supplementary appendix 1). Grey literature (including reports, conference and workshop proceedings) will be searched through Google scholar search engine and key relevant websites such as WHO African Index Medicus and African Journals Online (AJOL). Key individuals in the field will be contacted for any unpublished work and research papers that are under preparation. References will be exported and duplicates will be removed using any citation management software.

10.1136/bmjopen-2016-013540.supp1 supplementary appendix

### Selection of studies

The titles and abstracts of potentially relevant identified articles will be independently screened by two researchers for eligibility. Full-text copies of articles that will meet the eligibility criteria will be obtained. These full text articles will then be assessed by two independent researchers for inclusion in the review. Any disagreement about the eligibility will be resolved through discussion. A short questionnaire has been developed and used to guide the selection of relevant studies (see online supplementary appendix 2).

10.1136/bmjopen-2016-013540.supp2 supplementary appendix

### Quality assessment of included studies

The quality of all papers that will be included in the review will be assessed using the “Effective Public Health Practice Project quality assessment tool for quantitative studies”.^20^ Components assessed by the selected tool are selection bias, study design, confounders, blinding, data collection methods, withdrawals and attrition, intervention integrity and analyses (see online supplementary appendix 3). Quality assessment will be performed independently by two authors. Each paper will be carefully assessed and rated for selection bias, study design, confounders, blinding, data collection method and withdrawals and dropouts. A paper will be categorised as STRONG when there is no weak ratings for any of the listed components. A paper with one weak rating will be classified as MODERATE while with two or more weak ratings will be classified as WEAK. Discrepancies in rating between reviewers will be resolved by consensus.

10.1136/bmjopen-2016-013540.supp3 supplementary appendix

### Data extraction

This will be performed independently by two researchers. The following review characteristics and outcome data will be extracted from included studies using a standardised data form: study details (author, year of publication, country of study); study population (sample size, age range, sex distribution, number of children in intervention and control groups); intervention type, intervention characteristics (type, content, duration of study, follow-up time points, mode of delivery, intervention provider); study design; setting (urban–rural; private–public school); outcome data (changes in nutritional and physical activity knowledge, attitude and self-efficacy, increased intake of fruits and vegetables, lower consumption of high fat diets and sugar-sweetened beverages, increased participation in physical activity, changes in body weight or BMI-for-age); theoretical basis of intervention, potential confounders and key limitations of the study as specified by the authors. Additional information will be requested from authors where necessary.

### Data synthesis, assessing heterogeneity and publication bias

Data extracted will be summarised by country and region (Central Africa, Eastern Africa, Southern Africa, Northern Africa and Western Africa). Narrative synthesis will be used to summarise and explain the evidence for data that cannot be analysed using quantitative synthesis. This will be performed by grouping studies into thematic areas such as study designs, intervention characteristics and types and factors that influenced outcomes and programme implementation (facilitators and barriers). Summary statistics will be presented as frequencies, percentages, means, confident intervals and p values. Differences and similarities will be highlighted.

Estimates of effect sizes will be generated for each outcome across studies. The Grading of Recommendations Assessment, Development and Evaluation (GRADE) approach^21^ will be used to assess the overall quality and strength of evidence across studies. This will be performed by downgrading scores for a number of factors: study limitations, inconsistency of results, imprecision, reporting or publication bias and indirectness of evidence. Scores will be upgraded for studies with large effect sizes, dose–response gradient and confounders that are likely to minimise the effects. Final ratings for the quality of evidence of each outcome will be categorised as high, moderate, low or very low. The overall quality of the evidence will then be rated. Recommendations of the evidence on which school-based intervention programmes are effective in promoting healthy eating and physical activity behaviours of learners will be made considering the direction and strength of the evidence. The results will be summarised and presented in summary of findings tables.

Statistical heterogeneity across studies will be assessed with Cochran's Q statistic,^22^ and the I^2^ statistic^23^ will be used to determine the degree of heterogeneity between studies. To assess the potential sources of heterogeneity, subgroup analyses will be performed using the following variables: sex, age group, study setting (rural, peri-urban, urban; private–public school), criteria for classification of overweight and obesity, intervention types, intervention characteristics, geographical region (Central Africa, Eastern Africa, Southern Africa, Northern Africa and Western Africa), where data allow. Heterogeneity will also be tested by conducting meta-regression analysis. Funnel plots and Egger test of bias^24^ will be used to assess publication bias. Meta-analysis will be conducted for identical variables across studies such as study designs, data collection tools and intervention types, where data allow. Pooled estimates for the meta-analysis and their 95% CIs will be obtained using the random-effects model of DerSimonian–Laird.^25^ Studies will be weighted by the inverse of their variances. Where data allow, sensitivity analysis will be performed to assess robustness of the results by removing a study at a time and assessing the impact of I^2^ on the summary estimate. The inter-rater agreement for study inclusion and data extraction will be assessed using the Cohen's κ coefficient.^26^

Data analysis will be performed using the R statistical software (The R Foundation for statistical computing, Vienna, Austria).

## Presenting and reporting the review results

A PRISMA flow chart of search and study selection with included and excluded studies will be presented. Reasons for exclusion of studies will be given. Extracted data will be presented in tables. Summary statistics of quantitative data will be complemented with narrative syntheses. Quantitative data will be presented in tables of individual studies, summary tables and forest plots where appropriate. Outcome data will be examined by country (all 54 countries), region (Central Africa, Eastern Africa, Southern Africa, Northern Africa and Western Africa), sex, settings (urban, peri-urban, rural; private–public school), type and characteristics of intervention, study design and duration of study. The quality assessment and risk of bias scores determined for each included study will be presented in tables.

## Conclusions

This systematic review will summarise the current available evidence on characteristics, outcomes and effectiveness of school-based interventions targeted at improving diet and physical activity with the overall aim of reducing obesity prevalence and improving health. This attempt could identify potential factors that lead to successful interventions or barriers to the successful implementation of these programmes within the African context. This will inform the development of evidence-based interventions in the prevention and control of childhood obesity among African learners in African countries and to identify research gaps in the literature for further studies. These findings may also serve as a policy document to governments in designing effective programmes to curb the increasing prevalence of obesity and its associated health consequences. Moreover, application of the GRADE approach in rating the quality of evidence will strengthen the review. A limitation of this review is the likely exclusion of some potentially relevant studies that used other measures of body composition since only studies that using BMI and/weight changes as body composition changes will be included.

## Dissemination

All data that will be presented in this review are based on published articles. The findings of this systematic review will be submitted for publication in peer-reviewed journals and a chapter of a thesis. In addition, this will be disseminated in conferences and policy document to appropriate bodies for decision-making where needed.
