# Supplementary material for: Protocol for systematic review of school-based interventions to prevent and control obesity in African learners
Source: BMJ Open. 2017 Mar 27;7(3):e013540. doi: 10.1136/bmjopen-2016-013540 (PMC5372051; doi:10.1136/bmjopen-2016-013540)
Supplement: supplementary appendix [file bmjopen-2016-013540supp_appendix2.pdf]

Appendix 2: A short question guide for the selection of relevant studies based on inclusion criteria

No	Question	Action
1	Did the study use any of the eligible study designs?	Yes, move to next question<br>No, exclude
2	Did the study involve learners/schoolchildren aged 6-15 years?	Yes, move to next question<br>No, exclude
3	Were the study participants residing in any of the African countries?	Yes, move to next question<br>No, exclude
4	Did the study evaluate any of the following: nutrition interventions, physical activity interventions, combined nutrition and physical activity interventions, school environment?	Yes, move to next question<br>No, exclude
5	Were any of the following outcomes reported? Changes in nutrition and physical activity knowledge, attitude and self-efficacy, increased participation in physical activity, increased intake of fruits and vegetables, decreased consumption of high fat diets and sugar-sweetened beverages, changes in body weight or BMI-for-age; and reporting a baseline and a post-intervention measurements.	Yes, move to next question<br>No, exclude
6.	Were baseline and post-intervention measurements taken?	Yes, include study<br>No, exclude
